# The Role of CP Level and Interaction with Antibiotics in the Post-Weaning Piglets’ Diet: Growth Performance, Body Composition, Nutrient Digestion, and Intestinal Health

**DOI:** 10.3390/ani16010024

**Published:** 2025-12-21

**Authors:** Rui Wang, Lei Hou, Qiwen Wu, Xiaolu Wen, Yunxia Xiong, Xuefen Yang, Kaiguo Gao, Zongyong Jiang, Shuting Cao, Li Wang

**Affiliations:** 1 State Key Laboratory of Swine and Poultry Breeding Industry, Key Laboratory of Animal Nutrition and Feed Science in South China, Ministry of Agriculture and Rural Affairs, Guangdong Key Laboratory of Animal Breeding and Nutrition, Institute of Animal Science, Guangdong Academy of Agricultural Sciences, Guangzhou 510640, China; 2 Guangxi State Farms Yongxin Animal Husbandry Group Co., Ltd., Nanning 530022, China

**Keywords:** weaning piglets, crude protein levels, growth performance, body composition, intestinal health

## Abstract

The objective of this study is to evaluate the effects of crude protein (CP) levels and antibiotics in feed on the growth performance, body composition, nutrient digestion and absorption, gut health, and microbiota of weaned piglets. A total of 100 piglets (Duroc × Landrace × Yorkshire, weaned at day 21) with an initial weight of 6.39 ± 0.03 kg were randomly assigned to four treatments and fed with 18% CP antibiotic-free diets, 18% CP with antibiotics diets, 24% CP antibiotic-free diets, and 24% CP with antibiotics diets for 0–14 d (S1). Furthermore, the piglets were fed with an identical diet (19% CP and without antibiotics) starting day 15 until they reached 25 kg (S2). Results indicated that the average daily gain (ADG) and gain feed ratio (G:F) during the S1 phase as well as BW at day 14, were significantly decreased (*p* < 0.05) in the 18% CP group compared with the 24% CP group. Similarly, piglets in the 18% CP group showed a significantly decreased body protein content and body water, protein deposition rates at the S1 phase, and significantly improved the body lipid content and deposition rate but not (*p* > 0.05) at the S2 phase. The 18% CP group demonstrated significantly increased (*p* < 0.05) serum concentrations of Threonine, Valine, Isoleucine, and Lysine in weaned piglets during the S1 phase, while Glycine concentration significantly decreased, and no significant (*p* > 0.05) effects were observed in the S2 phase. Furthermore, dietary antibiotic supplementation had no effect on (*p* > 0.05) piglet growth performance, body composition, nutrient digestion, and metabolism throughout the entire trial period. In the absence of antibiotics, compared to the 24% CP diet group, the piglets fed with an 18% CP diet significantly increased the relative abundance of *Lactobacillus* in the colon of weaned piglets upon reaching 25 kg BW (*p* < 0.05). Compared to the diet without added antibiotics, the addition of antibiotics to the diet significantly decreased the relative abundance of *Pseudoramibacter* in the colon of weaned piglets on day 14. No significant (*p* > 0.05) difference was observed in intestinal morphology or gastrointestinal pH among the groups. In conclusion, lower dietary protein levels in the early post-weaning period reduced growth performance and promoted gut microbiota balance, and compensatory growth occurred after returning to higher protein levels two weeks after weaning.

## Simple Summary

This study highlights the critical interplay between CP levels and antibiotic use in shaping the growth and health of weaned piglets. The findings suggest that lower dietary protein (18% CP) might hinder growth performance temporarily but enables compensatory growth when protein levels are normalized. While the use of antibiotics did not enhance overall performance or body composition, it did modulate gut microbiota transiently. This implies that optimizing dietary protein could be a more sustainable and health-conscious strategy for piglet nutrition, reducing reliance on antibiotics in modern farming practices.

## 1. Introduction

Early-weaned piglets exhibit a high capacity for rapid growth and protein deposition, and therefore have high protein requirements. Protein, as a core nutrient for building body tissues and maintaining metabolic activities, directly affects a piglet’s growth performance [1,2]. Lower protein levels in diets reduced gastrointestinal burden and decreased harmful bacterial proliferation caused by undigested protein, thereby improving intestinal microecological balance [3]. However, protein levels that are too low may not meet the needs of piglets with high growth potential, limiting their growth rate [4]. On the other hand, previous studies suggested that diets containing approximately 22–24% CP may enhance Average Daily Gain (ADG) and Average Daily Feed Intake (ADFI) in early-weaned piglets when dietary amino acid balance and energy density were optimized [5]. However, this effect may vary with breed, sex, weaning age, weight, or amino acid profile. In addition, high protein intake may cause undigested protein to remain in the gastrointestinal tract, increasing ammonia emissions and intestinal health risks [6,7]. Based on this, it is essential to verify the extent of compensatory growth and protein deposition, specifically alongside the interaction with antibiotic withdrawal, when feeding piglets a diet with a protein level of 19% during the subsequent rearing phase.

Due to physiological immaturity, such as low disease resistance and underdeveloped digestive tracts, post-weaned piglets often experience ‘early weaning syndrome’ [8]. This syndrome is characterized by poor appetite, slow growth, diarrhea, and increased mortality [9]. To mitigate these issues, the swine industry has traditionally used in-feed antibiotics to reduce pathogenic bacterial load and prevent post-weaning diarrhea, thereby indirectly improving nutrient utilization and allowing for piglets to realize their growth potential [10]. However, the overuse of antibiotics can spread antibiotic-resistant bacteria and disrupt the intestinal microecology, posing threats to public health [11,12]. Consequently, there is a global regulatory trend toward restricting or banning antibiotic growth promoters. While total prohibition is the ultimate goal, immediate withdrawal without effective alternatives often leads to significant declines in performance and increased mortality rates [13]. To address this transition, a strategic reduction approach may be necessary. Therefore, the present study applied antibiotics strictly during the highly vulnerable S1 phase, then discontinued their use during the S2 phase. This design allows for investigation into the effects of this phased strategy on growth performance, body composition, and gut health in piglets.

The aim of this study is to investigate how early dietary protein restriction (18% and 24% CP) during the S1 phase, followed by restoration to 19% CP in the S2 phase, induces compensatory growth and enhances protein deposition efficiency without impairing intestinal integrity.

## 2. Materials and Methods

### 2.1. Animals and Experimental Design

**Figure 1:**
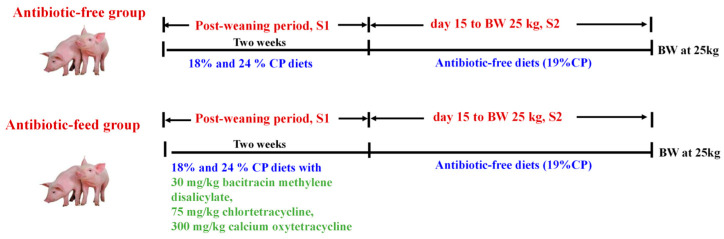
A schematic diagram of the experimental design. One hundred 21-day-old Duroc  ×  Landrace  ×  Yorkshire weaned piglets with an average initial body weight (BW) of 6.39  ±  0.03 kg were randomly divided into four groups: 18% CP antibiotic-free group, 18% CP with antibiotics group, 24% CP antibiotic-free group, and 24% CP with antibiotics group, with five replicates in each, comprising five piglets in each replicate. The entire experiment was divided into two phases: the S1 phase and the S2 phase. During the S1 phase, piglets in the antibiotic-free group were fed an 18% CP and 24% CP diet, and those in the antibiotic-feed group were fed an 18% CP and 24% CP diet supplemented with 30 mg/kg bacitracin methylene disalicylate, 75 mg/kg chlortetracycline, and 300 mg/kg calcium oxytetracycline for two weeks. During the S2 phase, all piglets were switched to the same antibiotic-free diet (19% CP) until they reached an average target BW of approximately 25 kg.

One hundred weaned piglets (21 days of age; 6.39 ± 0.03 kg BW; Duroc × Landrace × Yorkshire) were randomly assigned to 4 treatments: 18% CP antibiotic-free group, 18% CP with antibiotics group, 24% CP antibiotic-free group, 24% CP with antibiotics group with 5 animals per pen and 5 pens (3 pens with 3 barrows and 2 gilts, 2 pens with 2 barrows and 3 gilts) per group. The added antibiotics were 30 mg/kg bacitracin methylene disalicylate, 75 mg/kg chlortetracycline, and 300 mg/kg calcium oxytetracycline. During the restriction phase, the weaned piglets in different treatment groups were fed their respective experimental diets. Subsequently, from day 15 post-weaning until reaching 25 kg, all piglets were fed the same antibiotic-free diet (19% CP) until their average BW reached the average target BW of 25 ± 0.15 kg. The schematic diagram for the experimental design is shown in Figure 1. Experimental diets were formulated as pelleted feeds for piglets from 0 to 14 days post-weaning and from 15 days to 25 kg BW, based on a corn-soybean meal diet and formulated with reference to the NRC (2012) [14] nutrient requirements for 7–11 kg and 11–25 kg pigs. Ingredient composition and nutrient composition of the diets are presented in Table 1.

Piglets were housed in a nursery facility (2.20 × 1.50 m^2^), which had a hard plastic fully slatted floor, a multi-hole stainless feeder, and a single bowl drinker. Pigs had free access to feed and water throughout the experiment period.

### 2.2. Sample Collection

On day 0, day 14, and when the piglets reached approximately 25 kg, each piglet was weighed individually after a 12 h fast. The feed consumption of the piglets was recorded, and the Average Daily Gain (ADG), Average Daily Feed Intake (ADFI), and Gain-to-Feed (G:F) ratio were calculated for days 0–14 and from day 15 until the piglets reached 25 kg. On day 14 of the experiment, and when the piglets reached a BW of 25 kg, 10 mL of blood was collected from one piglet per pen (with BW close to the group average) via the anterior vena cava. The samples were centrifuged at 3500 r/min for 10 min to separate the serum, which was then transferred into sterile centrifuge tubes, labeled, and stored at −80 °C for later use. Fresh feces from each pen of piglets were collected for three consecutive days during the S1 phase (6–8 days post-weaning) and the S2 phase (27–29 days post-weaning) using the partial fecal collection method [15]. The samples were weighed, and for every 100 g of sample, 10 mL of 10% sulfuric acid was added and thoroughly mixed for nitrogen fixation. The treated samples were then stored at −20 °C for later use. On the 14th day after the start of the trial, 5 piglets were randomly selected from each group (two barrows and three gilts per treatment). After anesthesia with sodium pentobarbital, the piglets were slaughtered. The abdominal cavity was opened, and the stomach, small intestine, and large intestine were quickly ligated. The pH of the stomach, duodenum, jejunum, ileum, and colon contents was immediately measured using a portable pH meter (HI 9024C, HANNA Instruments, Woonsocket, RI, USA), with three repeated measurements taken and the average value recorded. The middle segment of the colon contents was collected in a sterile centrifuge tube and quickly frozen in liquid nitrogen, then stored at −80 °C for later use. Approximately 2 cm of the middle duodenum, jejunum, and distal ileum were taken, gently flushed with pre-cooled phosphate-buffered saline solution to remove contents, and then placed in 4% paraformaldehyde sample tubes for preservation.

Additionally, before the start of the experiment, five pigs (three barrows and two gilts) were selected as the initial slaughter group to measure baseline body composition. On day 14 of the experiment, and when the piglets reached 25 kg, following a 12 h fast and individual weighing, five piglets per group (three barrows and two gilts per treatment at each phase) with BW close to the group average were anesthetized with sodium pentobarbital and slaughtered by exsanguination, with all blood carefully collected from each piglet. The slaughter followed the procedure outlined by Hou et al. [16] and Jones et al. [17].

### 2.3. Serum Biochemical Parameter Measurement

Serum total protein, albumin, blood urea nitrogen, glucose, triglycerides, total cholesterol, alanine aminotransferase, aspartate aminotransferase, alkaline phosphatase, and creatinine levels were determined using an automated biochemical analyzer (Selectra Pro XL, Vital Scientific, Woerden, The Netherlands) according to the manufacturer’s instructions. All reagent kits were purchased from Sinopharm Beijing Bio-Technology Co., Ltd, located in Beijing, China.

### 2.4. Serum Free-Amino-Acid Concentration Determination

Briefly, 0.4 mL of the serum sample was accurately pipetted into a sterile centrifuge tube. Then, 1.2 mL of 10% sodium sulfosalicylate was added to the precipitate sample proteins. After thorough vortexing, the sample was centrifuged at 12,000 r/min for 15 min at 4 °C. Subsequently, the supernatant was collected and filtered through a 0.22 μm aqueous phase filter before analysis. Free amino acid concentrations in the serum were determined using an automated amino acid analyzer (Hitachi, Tokyo, Japan) based on the principle of post-column ninhydrin derivatization.

### 2.5. Feed and Fecal Nutrients Determination

Water content was determined by drying to constant weight in an oven at 103 °C ± 2 °C; CP content was estimated by multiplying the total nitrogen content, determined using a Kjeltec 8400 analyzer (FOSS Analytical AB, Höganäs, Sweden), by a factor of 6.25. The ether extract content was determined using an automated extraction analyzer (Ankom Technology, Macedon, NY, USA). Ash content was determined by incineration in a muffle furnace at 550 °C to constant weight. Gross energy content of the diets and fecal samples was determined using an oxygen bomb calorimeter (Parr Instrument Company, Moline, IL, USA). Furthermore, the amino acid content in the feed was measured by hydrolyzing with 6 mol/L hydrochloric acid at 110 °C for 24 h, and then analyzing the sample using a fully automatic amino acid analyzer (Hitachi, Tokyo, Japan) based on the post-column derivatization principle with the indanone method.

### 2.6. Body Composition Measurement

The dry matter, CP, ether extract, ash, and gross energy content of the whole empty body were determined as described in the feed and fecal nutrients determination. By dividing the differences in body composition content by the corresponding experimental days, the daily deposition of water, protein, lipid, and ash (g/d) in piglets was calculated, as follows:

body water deposition (g/d) = [(body water percentage final BW) − (body water percentage initial BW)]/(corresponding trial day)

### 2.7. Intestinal Morphology Measurement

The intestinal segments fixed in 4% polyformaldehyde solution were processed through trimming, washing, dehydration, wax immersion, embedding, sectioning, and hematoxylin-eosin staining. The tissue samples were cut into sections of 3 µm thickness. To ensure representative sampling and avoid resampling the same structure, 5 serial sections were skipped between each evaluation. A total of 10 intact villi and crypts were measured per section using the Case Viewer image analysis software (3DHISTECH, CaseViewer2.2, Budapest, Hungary).

### 2.8. Colon Gut Microbiota

According to the method by He et al. [18], total genomic DNA was extracted from the colon contents using the QIAamp PowerFecal DNA Kit (Qiagen, Hilden, Germany). The V3–V4 hypervariable regions of the 16S rRNA gene were amplified using primers 338F (5′-ACTCCTACGGGAGGCAGCA-3′) and 806R (5′-GGACTACHVGGGTWTCTAAT-3′). Sequencing libraries were generated using the TruSeq^®^ DNA PCR-Free Sample Preparation Kit and sequenced on the Illumina NovaSeq platform. Raw reads were quality-filtered based on Q20/Q30 scores, and chimeric sequences were removed to obtain effective tags. These valid sequences were clustered into operational taxonomic units (OTUs) at 97% identity using Uparse (v7.0.1001). Taxonomic annotation was performed using the Mothur method against the SILVA 138 database, while phylogenetic relationships were analyzed using MUSCLE (Version 3.8.31). Finally, data were normalized to the minimum sample depth for α- and β-diversity calculations.

### 2.9. Statistical Analysis

Data analysis was performed by two-way ANOVA using SPSS 20 software to analyze the general univariate linear model, determining the main effects of CP treatment, antibiotic treatment, and their interaction. For parameters showing a significant interaction effect (*p* < 0.05), an Analysis of Variance (ANOVA) was performed, and the means were separated using Duncan’s multiple range test at *p* < 0.05 significance. Prism 8.0 software was used for colonic microbiota analysis and visualization. Statistical significance was declared at *p* < 0.05, and tendencies were declared at 0.05 < *p* < 0.10. Data were presented as means ± SEM.

## 3. Results

### 3.1. Growth Performance

Performance data during the S1 and S2 phases are summarized in Table 2. The ADG and G:F during the S1 phase, as well as BW at day 14, were significantly decreased in the 18% CP groups compared with the 24% CP groups. During the S2 phase, piglets offered the 18% CP diet exhibited a significant improvement in ADFI (*p* < 0.05). However, when assessed over the whole trial, the 18% CP treatment produced a significant reduction in G:F (*p* < 0.05). However, the antibiotics demonstrated no significant difference (*p* > 0.05) in ADG, ADFI, and G:F among the piglet groups across the overall experiment. Additionally, this study also found that there was no significant (*p* > 0.05) interaction between CP levels and antibiotics on the ADG, ADFI, and G:F of piglets.

### 3.2. Body Composition Content

As indicated in Table 3, the weaned piglets fed an 18% CP diet exhibited significantly decreased (*p* < 0.05) body protein content and amount of retained energy as protein on day 14, while substantially increasing (*p* < 0.05) body ash content, body lipid content, retained energy as lipid, the ratio of body lipid to body protein, and the ratio of body ash to body protein. Furthermore, both the analyzed gross energy and the calculated gross energy were significantly higher compared to the 24% CP groups (*p* < 0.05). Compared with diets not containing antibiotics, antibiotic supplementation significantly decreased (*p* < 0.05) the amount of retained energy as lipid in weaned piglets on the S1 phase. However, no significant differences (*p* > 0.05) were detected in body water, body protein, body lipid, body ash, the ratio of body water to body protein, the ratio of body lipid to body protein, the ratio of body ash to body protein, analyzed gross energy, calculated gross energy, or retained energy as protein. Furthermore, a significant interaction (*p* < 0.05) between dietary CP level and antibiotic supplementation was observed for body protein, the ratio of body water to body protein, and retained energy as protein in weaned piglets on day 14 of the S1 phase.

According to the data in Table 4, dietary CP levels and antibiotics during the S1 phase had no significant (*p* > 0.05) effects on body water, body protein, body lipid, body ash, the ratio of body water to body protein, the ratio of body lipid to body protein, the ratio of body ash to body protein, analyzed gross energy, calculated gross energy, retain energy as protein, and retain energy as lipid when piglets reached 25 kg.

### 3.3. Body Composition Deposition Rates

Body composition deposition data during the S1 phase are summarized in Table 5. Compared to the 24% CP group, the 18% CP group significantly reduced (*p* < 0.05) body water and protein deposition rates while significantly improving (*p* < 0.05) body lipid deposition rate in weaned piglets during the S1 phase. Compared to the 24% CP group, 18% CP levels from 0 to 14 days of age tended to significantly improve (0.05 < *p* < 0.10) body water deposition and protein deposition rate in piglets at 25 kg BW (Table 6). In addition, compared to the group without antibiotics, the addition of antibiotics from the S1 phase, S2 phase, and whole experimental phase had no significant (*p* > 0.05) effect on the deposition rates of body water, body protein, body lipid, body ash, and gross energy in piglets.

Moreover, this study found an interaction effect (*p* < 0.05) between CP levels and antibiotics on protein and gross energy deposition rate in weaned piglets during the S1 phase. Additionally, an interaction (*p* < 0.05) was observed between dietary CP levels and antibiotic administration on body lipid and gross energy deposition rates in the S2 phase. However, during the phase from day 0 to 25 kg BW, protein levels and antibiotics had no significant effects (*p* > 0.05) on water, protein, lipid, ash, or gross energy deposition rates (Table 6). Notably, an interactive effect (*p* < 0.05) of CP levels and antibiotics on lipid deposition rate was identified during this same period (Table 7).

### 3.4. Serum Biochemistry

The serum biochemical parameters in weaned piglets are presented in Table 8. During the S1 phase with 18% CP diet, weaned piglets showed significantly decreased (*p* < 0.05) serum blood urea nitrogen (BUN) levels and significantly increased (*p* < 0.05) serum Triglyceride (TG) levels at day 14, while serum total protein (TP) and glucose (GLU) levels demonstrated a significant upward trend (0.05< *p* < 0.10). Compared to feed without antibiotics, the addition of antibiotics had no effect (*p* > 0.05) on serum biochemical parameters of piglets in the S1 and S2 phases (Table 8 and Table 9).

Additionally, this study found no significant interaction effects (*p* > 0.05) between dietary CP levels and antibiotics in the S1 and S2 phase on serum biochemistry in piglets (Table 8 and Table 9).

### 3.5. Apparent Digestibility of Nutrients

As shown in Table 10, compared with the 24% CP group, the 18% CP group tended to decrease the apparent digestibility of CP (0.05< *p* < 0.10), but exhibited no significant differences (*p* > 0.05) in dry matter, ether extract, ash, and gross energy. During the S2 phase, the 18% CP group significantly decreased (*p* < 0.05) the apparent digestibility of CP and gross energy in piglets (Table 11). The addition of antibiotics to the diet during the S1 period significantly decreased (*p* < 0.05) the apparent digestibility of ash in weaned piglets, but the antibiotics had no significant (*p* > 0.05) effect on dry matter, ether extract, CP, and gross energy.

This study found that the apparent digestibility of nutrients was not influenced by CP level and antibiotics in diets of weaning piglets.

### 3.6. Serum Free Amino Acid Concentration

Table 12 shows that during the S1 phase, the 18% CP level diets resulted in significantly increased (*p* < 0.05) serum concentrations of Threonine, Valine, Isoleucine, Lysine, and Glycine in weaned piglets (*p* < 0.05). During the S2 phase, 18% CP dietary significantly improved serum Serine concentrations in piglets weighing 25 kg (*p* < 0.05). Additionally, supplementing antibiotics during the S1 phase also led to a significant increase (*p* < 0.05) in serum Glycine concentration in weaned piglets, but not (*p* > 0.05) at the S2 phase (Table 12 and Table 13).

There was a significant interaction (*p* < 0.05) between different levels of CP and the addition of antibiotics on the serum Glycine and Arginine concentrations of weaned piglets on day 14 during the S1 phase, but not (*p* > 0.05) at the S2 phase (Table 12 and Table 13).

### 3.7. Small Intestinal Morphology and Gastrointestinal Content pH

Neither the 18% nor 24% dietary CP levels during the S1 period, nor the addition of antibiotics, had a significant (*p* > 0.05) effect on villus height, crypt depth, or villus height-to-crypt depth ratio in the duodenum, jejunum, and ileum of piglets at day 14 post-weaning (Table 14).

This study found that dietary CP level, with or without antibiotics, had no significant (*p* > 0.05) effect on the pH of gastric, duodenal, jejunal, ileal, and colonic contents of weaned piglets on day 14 (Table 15).

### 3.8. Colonic Microbiota Alpha Diversity and Genera

**Figure 2:**
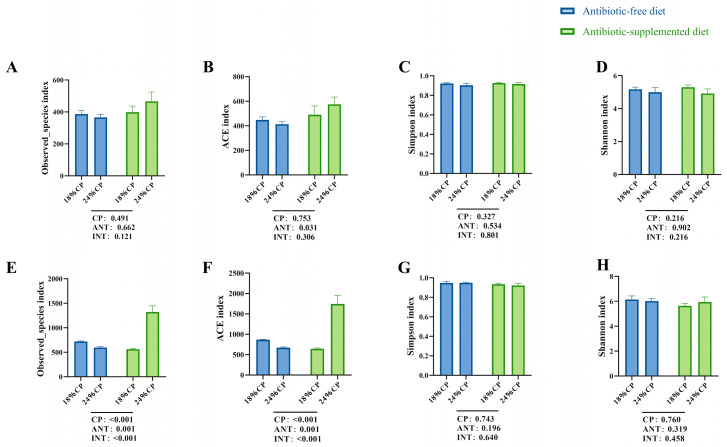
Effects of with or without antibiotics feed and CP levels on colonic microbiota in weaned piglets. The *α*-diversity indices, including Observed_species, Ace, Shannon, and Simpson, at the S1 phase (**A**–**D**) and the S2 phase (**E**–**H**). Data are expressed as means ± SEM. CP—main effect of crude protein; ANT—main effect of antibiotic; INT—interaction effect between crude protein and antibiotic.

In the S1 phase, antibiotic supplementation had a significant impact (*p* < 0.05) on the ACE index (Figure 2B). In the S2 phase, both CP level and antibiotic supplementation, as well as their interaction, significantly affected (*p* < 0.05) the observed species and ACE index (Figure 2E,F). Specifically, antibiotic supplementation at the 24% CP level led to a substantial increase in these indices. The Simpson and Shannon indices were not significantly affected (*p* > 0.05) by CP levels, antibiotic supplementation, or their interaction in either phase (Figure 2C,D,G,H).

**Figure 3:**
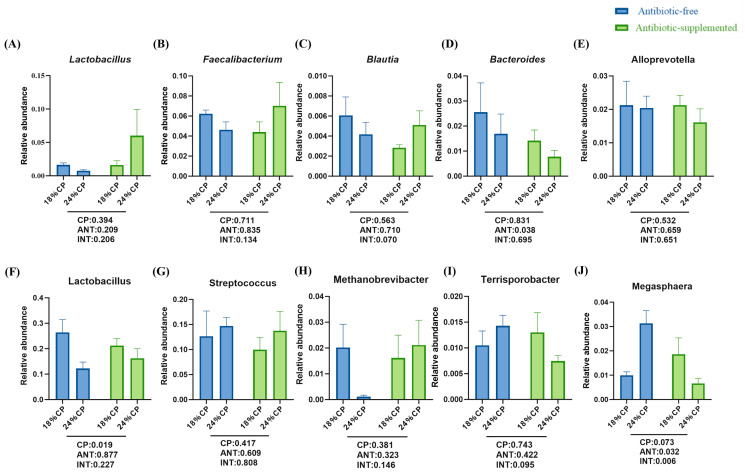
Effects of with or without antibiotics feed and CP levels on colonic microbiota in the S1 phase (**A**–**E**) and the S2 phase (**F**–**J**). Data are expressed as means ± SEM. CP—main effect of crude protein; ANT—main effect of antibiotic; INT—interaction effect between crude protein and antibiotic.

In phase S1, feeding antibiotic-supplemented diets significantly decreased the relative abundance of *Bacteroides* in the colon of weaned piglets on day 14 (Figure 3D) (*p* < 0.05). However, after antibiotic withdrawal in phase S2, it significantly decreased the relative abundance of *Megasphaera* (Figure 3J) in the colon of piglets at 25 kg BW (*p* < 0.05). Under antibiotic-free conditions, the 24% or 18% levels of CP in the S1 diet had no significant effect on the colon microbiota of weaned piglets from 0 to 14 days. However, in phase S2, after uniformly feeding a 19% CP diet, the 18% CP group in S1 significantly increased the relative abundance of *Lactobacillus* in the colon of piglets at 25 kg BW (*p* < 0.05).

## 4. Discussion

This study found that a dietary CP level of 18% significantly decreased the growth performance of piglets two weeks post-weaning compared to a 24% CP diet, consistent with the findings from Wellock et al. [19]. Additionally, this study found that although the 18% CP level groups from 0 to 14 days reduced the growth performance of weaned piglets, after restoring the feed to a 19% CP level during the S2 phase, the piglets exhibited some compensatory growth. Therefore, there was no difference in the number of experimental days and ADG from 0 to 25 kg among the piglet groups. Stein et al. investigated the effects of feeding weaned piglets diets containing either 20.8% or 15.7% CP from days 0 to 14, followed by either 17.5% or 19.3% CP from days 15 to 35 [20]. They found that piglets receiving the 15.7% CP diet from days 0 to 14 exhibited reduced growth performance, but their growth was compensated when fed the 19.3% CP diet from days 15 to 35. Similarly, Shi et al. reported that piglets experiencing early-stage protein restriction, followed by restoration to normal protein levels, underwent compensatory growth [21]. The findings of the present study were consistent with those previously reported. This study found that dietary antibiotic supplementation did not significantly improve the growth performance of weaned piglets, consistent with the findings of Holt et al. [22], but inconsistent with those of Diana et al. [23], Kyriakis et al. [24], and Weber et al. [25]. Holt et al. [22] found that antibiotics had little effect on the growth performance of piglets reared in clean, isolated facilities with high labor input. The positive effects of antibiotics were usually associated with their ability to suppress the growth of certain pathogenic microorganisms [10]. The lack of significant improvement in growth performance with antibiotic supplementation observed in this study may be attributed to the high sanitary conditions of the experimental environment. The piglets were housed in a controlled research facility with strict biosecurity measures, regular cleaning protocols, and optimal environmental management. 

The early stage after weaning is considered one of the most effective periods for converting nutrients into animal tissue [26]. As expected, the body composition of piglets in the first two weeks after weaning was closely related to the CP level in the feed. With the increase in CP level in the feed, the protein content, protein deposition rate, and protein deposition energy in the piglets’ bodies increased, which was consistent with the research results from Conde-Aguilera et al. [27] and Ruiz-Ascacibar et al. [28]. The protein deposition rate was mainly determined by the pig’s lean meat growth potential [29], which suggested that the 18% CP supply in this study may have limited the pig’s genetic potential, despite the amino acid requirements being met. Therefore, it was not only necessary to ensure the required amino acid needs, but also to guarantee the protein (total nitrogen) needs to achieve the pig’s maximum protein deposition capacity. This study also found that with the increase in CP level in the feed, the ratio of body lipid to body protein decreased significantly, and the body lipid content, body lipid deposition rate, and body lipid energy decreased. Morazán et al. [30] and Skiba et al. [31] reported that pigs fed a low-protein, balanced, amino acid feed were more obese than those fed high-protein feed. This phenomenon may be due to the low protein deposition rate in animals fed low-protein feed, resulting in a large amount of energy being stored as body lipid. While the protein deposition rate increased with the increase in CP level in the feed, the energy stored as body lipid decreased [32]. Studies have shown that body water or body ash content is closely related to body protein content, reflecting the accompanying changes in body protein [33,34]. Therefore, the ratio of body water to body protein or the ratio of body ash to body protein should remain constant under different CP treatments. This study found that increasing the CP level in the feed did not significantly affect the ratio of body water to body protein, but significantly decreased the ratio of body ash to body protein. Oresanya et al. [35] also found that the body water–protein ratio was not affected by the feed level, but the ratio of body ash to protein decreased with the increase in feed level, and speculated that it was due to the faster protein deposition rate than the body ash deposition rate, resulting in a decrease in the ratio of body ash to protein. The results of this study were consistent with those of Oresanya et al. [35], as the piglets in this study had a high protein deposition capacity and had not yet reached their protein deposition limit. This study found that although reducing the CP level from 0 to 14 days decreased the protein deposition rate of weaned piglets, there was no difference in the protein deposition rate from 0 to 25 kg among the groups after the feed was restored to a normal CP level from 15 days to 25 kg. This suggested that piglets fed low-protein feed may have undergone compensatory protein deposition in the later stage. Bikker et al. [36], Carstens et al. [37], and Drouillard et al. [38] all reported that compensatory protein deposition occurred after a period of nutritional restriction, and the results of this study were consistent with theirs. Additionally, the present study revealed no significant impact of dietary antibiotics on body composition and deposition rates in weaned piglets at both day 14 and 25 kg BW.

Apparent digestibility of nutrients reflects the capacity of animals to digest and absorb nutrients from their diet. Greater digestive and absorptive capacity is beneficial for animal growth [39]. Bikker et al. [6] reported that piglets fed a 22% CP diet had higher apparent CP digestibility than those fed a 15% CP diet, but apparent ether extract digestibility was not significantly affected. These findings were largely consistent with the present study, where piglets fed a 24% CP diet had higher apparent CP and gross energy digestibility than those fed an 18% CP diet, with no significant effect on apparent ether extract digestibility. The apparent nutrient digestibility results from these studies indicated that apparent nutrient digestibility in piglets improved with age and also reflected the relatively low apparent CP digestibility in piglets immediately post-weaning, which explained why low-protein diets were used for weaned piglets at higher risk of diarrhea.

Serum urea nitrogen can serve as an indicator of dietary protein quality and animal nitrogen intake, as well as a response parameter for determining animal protein requirements [40]. This study found that feeding 24% levels of CP increased serum urea nitrogen content in weaned piglets, which was consistent with previous reports [39,41,42]. This may be due to the increased absorption and breakdown of CP in weaned piglets, leading to an increase in serum urea nitrogen content. Serum triglyceride and cholesterol content can reflect the lipid metabolism status of animals [43]. This study found that 24% CP levels decreased serum triglyceride content, indicating that increasing dietary CP levels reduced lipid synthesis. Morise et al. [44] reported that piglets consuming less protein between 7 and 28 days of age increased lipid deposition. Morazán et al. [30] and Ruusunen et al. [29] also reported that pigs fed low-protein diets were more obese compared to those fed normal-protein diets. This observation aligned with the protein leverage hypothesis, which suggested that animals prioritize meeting their protein requirements; consequently, when fed low-protein diets, they tended to overconsume total energy to reach their protein target, leading to increased fat accumulation [45,46]. Furthermore, the surplus energy not utilized for protein synthesis was repartitioned towards lipogenesis. Additionally, this study found that adding antibiotics to the feed did not affect serum urea nitrogen, triglyceride, and cholesterol content in weaned piglets. Furthermore, this study found that early dietary CP levels and antibiotics did not have a significant impact on subsequent serum biochemical parameters in piglets at 25 kg, which was consistent with the results of subsequent growth performance.

The concentration of free amino acids in serum can reflect the physiological condition and nutritional status of animals [43]. Serum amino acid concentration is influenced by factors such as protein synthesis and degradation, amino acid transport and metabolism, and gut microbiota structure, and is an intermediate of the body’s metabolic process [47,48]. Proteins in feed are hydrolyzed into amino acids under the action of proteases and peptidases secreted by the host and gut microbiota, and are then absorbed by amino acid transporters in the gut. This study found that despite the consistent levels of lysine, threonine, valine, glycine, and isoleucine in each feed group, the 24% CP level in feed significantly decreased the serum concentrations of threonine, valine, isoleucine, glycine, and lysine in weaned piglets. The reason for this phenomenon may be that the increased CP level in feed reduced the amount of crystalline amino acids added, and the absorption rate of crystalline amino acids was much higher than that of amino acids produced by protein degradation. Meanwhile, this study also found that providing antibiotics in the diet from days 0 to 14 significantly increased glycine concentration but did not have a significant impact on subsequent serum amino acid concentrations when weaned piglets reached a BW of 25 kg. These findings were consistent with the research results reported by Yu et al. [49].

The gastrointestinal tract is the primary site for the digestion and absorption of nutrients in animals, and its pH significantly affects the normal physiological function. A suitable acidic environment in the gastrointestinal tract not only ensures the activity of digestive enzymes, promotes the digestion and absorption of nutrients, but also improves the balance of the microbial community [50]. Wellock et al. reported that the pH of the contents in the ileum, cecum, proximal colon, and distal colon of piglets 14 days after weaning was not affected by the level of crude protein in the diet (13%, 18%, and 23%) [19]. Htoo et al. also reported that feeding piglets with high or low crude protein diets did not affect the pH of the contents in the ileum and cecum [5]. This study found that the pH of the gastrointestinal tract contents in weaned piglets was not affected by the level of CP in the diet with or without antibiotics, which was consistent with their results. The morphological characteristics of the small intestine are an important indicator of the integrity of the intestinal mucosa and its absorptive function in pigs [51]. The villi and crypts in the small intestine are tubular structures formed by the migration of epithelial cells towards the intestinal lumen, and their structure directly affects the digestion and absorption of nutrients [52]. A decrease in villus height and an increase in crypt depth indicate atrophy of the intestinal mucosa and a decrease in absorptive capacity. Therefore, the villus height to crypt depth ratio is often used to reflect the functional status of the small intestine. Pearce et al. [53] reported that feeding 17% CP and 24% CP diets to weaned piglets had no significant effect on the villus height, crypt depth, and villus height to crypt depth ratio in the small intestine, which was consistent with the results of this study.

The gut microbiota and its metabolites play a crucial role in animal health, and feed is one of the main factors influencing the composition of the gut microbiota [16,54,55]. Therefore, this study investigated the effects of different CP levels in feed with or without antibiotics on the gut microbiota of weaned piglets. In the absence of antibiotics, this study found that compared to feed with 24% CP, the addition of 18% CP significantly improved the relative abundance of beneficial *Lactobacillus* in the colon of weaned piglets at 25 kg BW, but not at 0–14 d. Lactobacillus is a beneficial bacterium that can help maintain gut health and enhance immune system function [56]. This study found that compared to the diet without added antibiotics, the addition of antibiotics from day 0 to 14 significantly decreased the relative abundance of *Pseudoramibacter* in the colon of weaned piglets on day 14. However, after stopping the use of antibiotics from day 15 to 25 kg, there was a significant increase in the relative abundance of *Megasphaera* in the colon of piglets at 25 kg. In the context of the porcine gut, *Megasphaera* is functionally important as a lactate-utilizing bacterium that produces short-chain fatty acids (SCFAs), particularly butyrate and valerate [57]. The increase in its abundance suggested a recovery of the gut’s capacity to convert lactate into beneficial metabolites following antibiotic withdrawal. The results of this study indicated that feeding high levels of CP during the S1 phase reduced the abundance of beneficial bacteria. Feeding antibiotics during the S1 phase reduced the production of harmful bacteria, while stopping antibiotics in the subsequent S2 phase increased the abundance of harmful bacteria.

## 5. Conclusions

In conclusion, optimizing dietary CP levels appeared to be a more critical strategy for weaned piglets than antibiotic supplementation, which showed only transient microbiota changes without improving growth. However, while the 18% CP diet allowed for compensatory weight gain, it significantly increased lipid deposition. Consequently, the practical application of low-protein diets requires a careful economic trade-off between reduced feed costs and the potential risk of compromised carcass leanness at slaughter.

## Figures and Tables

**Table 1:** Ingredient and nutrient composition of experimental diets in piglets (% as-fed basis).

Item	S1 Phase (day 0 to 14)		S2 Phase (day 15 to 25 kg of BW)
	18% CP	24% CP	19% CP
Ingredients, %			
Corn	38.59	28.34	68.43
Expanded corn	10.00	10.00	–
Expanded soybean	8.00	8.00	–
Soybean meal, enzyme-treated	10.00	10.00	10.00
Soybean meal	1.05	5.88	14.46
Fish meal	2.00	5.88	2.00
Whey protein concentrate	2.00	5.88	–
Whey powder, 3.0%CP	15.00	15.00	–
Yeast extract	2.00	2.00	–
Soybean hulls	2.00	2.00	–
Soybean oil	1.00	1.00	1.00
Sucrose	2.50	2.50	–
Salt	0.20	0.20	0.35
Dicalcium phosphate	1.32	0.50	0.55
Calcium citrate	0.91	0.84	–
Limestone	–	–	0.93
L-Lys HCl	0.81	0.17	0.47
DL-Met	0.15	0.01	0.06
L-Thr	0.29	–	0.12
L-Trp	0.06	–	0.01
L-Val	0.22	–	–
L-Ile	0.10	–	–
Phytase	0.02	0.02	0.02
Zinc oxide	0.18	0.18	–
Choline chloride	0.20	0.20	0.20
Titanium dioxide	0.40	0.40	0.40
Premix ^1^	1.00	1.00	1.00
Total	100.00	100.00	100.00
Calculated nutrient composition ^2^, %			
NE ^3^, MJ/kg	10.88	10.78	10.50
SID ^4^ Lys	1.41	1.41	1.23
SID ^4^ Met	0.41	0.41	0.36
SID ^4^ Thr	0.83	0.84	0.73
SID ^4^ Trp	0.23	0.27	0.20
SID ^4^ Val	0.89	1.00	0.78
SID ^4^ Ile	0.72	0.95	0.71
SID ^4^ Leu	1.27	1.80	1.51
SID ^4^ Phe	0.65	0.89	0.83
SID ^4^ Arg	0.83	1.16	1.11
SID ^4^ His	0.35	0.49	0.45
Calcium	0.82	0.82	0.75
Total phosphorus	0.72	0.72	0.61
Analyzed nutrient composition, %			
CP ^5^	18.18	23.79	19.35

Abbreviations: ^1^ Provided, per kilogram of diet, 12,400 IU vitamin A, 2800 IU vitamin D3, 30 mg vitamin E, 5 mg vitamin K3, 3 mg thiamin, 10 mg riboflavin, 40 mg niacin, 8 mg pyridoxine, 40 μg vitamin B12, 0.08 mg biotin, 15 mg pantothenic acid, 1 mg folic acid, 80 mg Zn, 120 mg Fe, 70 mg Mn, 16 mg Cu, 0.7 mg I, and 0.48 mg Se. ^2^ Values were calculated according to NRC (2012) [14]. ^3^ NE—net energy. ^4^ SID—standardized ileal digestible. ^5^ CP—crude protein.

**Table 2:** Effects of CP level on growth performance of piglets fed diets with or without antibiotics.

Item	Antibiotic-Free Diet		Antibiotic-Supplemented Diet		SEM	*p*-Value		
	CP Level (%)		CP Level (%)					
	18	24	18	24		CP	ANT	INT
S1, post-weaning period (day 0 to 14)								
ADG, g/d	273.43	365.09	322.49	355.89	10.6	0.001	0.189	0.062
ADFI, g/d	372.33	423.69	417.33	420.07	8.97	0.121	0.228	0.160
Gain to feed	0.73	0.86	0.77	0.85	0.01	<0.001	0.463	0.123
Diarrhea rate (%)	1.72	0.86	0.29	2.29	0.37	0.436	0.999	0.063
S2, nursery period (day 15 to 25 kg BW)								
Final BW, kg	24.73	25.33	25.02	24.88	0.21	0.618	0.864	0.431
ADG, g/d	552.97	499.01	526.53	510.54	10.97	0.131	0.738	0.399
ADFI, g/d	898.50	818.66	859.03	797.35	15.73	0.025	0.298	0.752
Gain to feed	0.62	0.61	0.61	0.64	0.01	0.516	0.498	0.413
Diarrhea rate (%)	5.25	1.94	4.76	4.28	0.53	0.068	0.356	0.165
Overall (day 0 to 25 kg BW)								
Experimental days, d	40.00	41.80	40.75	40.60	0.33	0.212	0.727	0.144
ADG, g/d	458.61	453.88	458.06	456.72	7.21	0.852	0.944	0.917
ADFI, g/d	716.08	685.73	708.61	666.74	11.00	0.124	0.558	0.798
Gain to feed	0.64	0.66	0.65	0.69	0.01	0.040	0.284	0.507
Diarrhea rate (%)	3.59	1.45	2.67	3.35	0.35	0.260	0.448	0.040

Abbreviations: CP—main effect of crude protein; NT— main effect of antibiotic; INT—interaction effect between crude protein and antibiotic. Statistical significance was declared at *p* < 0.05, and tendencies were declared at 0.05 < *p* < 0.10.

**Table 3:** Effects of CP level on body composition of weaned piglets fed diets with or without antibiotics.

Item		Antibiotic-Free Diet		Antibiotic-Supplemented Diet		SEM	*p*-Value		
		CP Level (%)		CP Level (%)					
	ISG ^1^	18	24	18	24		CP	ANT	INT
Water, g/kg	696.75	705.41	706.37	698.44	716.3	2.45	0.052	0.741	0.077
Protein, g/kg	156.09	155.54 ^b^	166.15 ^a^	160.58 ^ab^	160.91 ^ab^	1.32	0.019	0.962	0.026
Lipid, g/kg	108.13	106.6	91.98	100.90	87	2.28	0.001	0.120	0.914
Ash, g/kg	30.42	30.66	27.99	29.95	28.33	0.43	0.014	0.815	0.501
Water: protein	4.46	4.54 ^a^	4.25 ^b^	4.35 ^ab^	4.49 ^a^	0.04	0.283	0.755	0.007
Lipid: protein	0.69	0.69	0.55	0.63	0.55	0.02	<0.001	0.093	0.164
Ash: protein	0.19	0.20 ^a^	0.17 ^b^	0.19 ^a^	0.18 ^ab^	0	0.004	0.672	0.039
Analyzed gross energy ^2^, MJ/kg	8.05	7.89	7.64	7.96	7.25	0.09	0.005	0.293	0.124
Calculated gross energy ^3^, MJ/kg	7.98	7.91	7.58	7.80	7.26	0.08	0.007	0.139	0.444
Retain energy as protein ^3^, MJ/kg	3.70	3.67 ^c^	3.94 ^a^	3.80 ^b^	3.79 ^bc^	0.03	0.006	0.863	0.003
Retain energy as lipid ^3^, MJ/kg	4.28	4.28	3.64	3.99	3.48	0.09	<0.001	0.056	0.601

Abbreviations: CP—main effect of crude protein; ANT—main effect of antibiotic; INT—interaction effect between crude protein and antibiotic. Data were subjected to two-way ANOVA. When the interaction was significant, treatment means were compared using Duncan’s multiple range test. ^a,b,c^ Means within a row with different superscripts differ significantly (*p* < 0.05). ^1^ ISG—initial slaughter group, whose data were used to estimate the initial body composition and were not included in the statistical analysis. ^2^ Analyzed by oxygen bomb calorimeter. ^3^ Calculated from analyzed body protein and lipid content by using 0.0237 MJ/g and 0.0396 MJ/g for retained protein and lipid energies, respectively. Statistical significance was declared at *p* < 0.05, and tendencies were declared at 0.05 < *p* < 0.10.

**Table 4:** Effects of S1 phase diets with or without antibiotics and CP level on subsequent body composition of piglets.

Item	Antibiotic-Free Diet		Antibiotic-Supplemented Diet		SEM	*p*-Value		
	CP Level (%)		CP Level (%)					
	18	24	18	24		CP	ANT	INT
Water, g/kg	690.18	698.15	695.85	689.62	2.64	0.879	0.802	0.226
Protein, g/kg	165.39	169.25	164.5	163.19	0.94	0.463	0.059	0.149
Lipid, g/kg	107.78	100.26	102.84	112.6	2.66	0.835	0.497	0.127
Ash, g/kg	26.96	26.37	27.05	28.17	0.43	0.771	0.302	0.344
Water: protein	4.17	4.13	4.21	4.23	0.03	0.813	0.235	0.535
Lipid: protein	0.65	0.59	0.64	0.69	0.02	0.983	0.205	0.147
Ash: protein	4.17	4.13	4.21	4.23	0.03	0.813	0.235	0.535
Analyzed gross energy ^1^, MJ/kg	8.29	8.04	7.91	8.41	0.11	0.576	0.985	0.105
Calculated gross energy ^2^, MJ/kg	8.22	7.98	6.98	8.32	0.26	0.298	0.394	0.144
Retain energy as protein ^2^, MJ/kg	3.95	4.01	3.12	3.87	0.22	0.381	0.289	0.451
Retain energy as lipid ^2^, MJ/kg	4.27	3.97	4.07	4.46	0.11	0.838	0.498	0.128

Abbreviations: CP—main effect of crude protein; ANT—main effect of antibiotic; INT—interaction effect between crude protein and antibiotic. ^1^ Analyzed by oxygen bomb calorimeter. ^2^ Calculated from analyzed body protein and lipid content by using 0.0237 MJ/g and 0.0396 MJ/g for retained protein and lipid energies, respectively. Statistical significance was declared at *p* < 0.05, and tendencies were declared at 0.05 < *p* < 0.10.

**Table 5:** Effects of CP level on body composition deposition rates of weaned piglets fed diets with or without antibiotics.

Item	Antibiotic-Free Diet		Antibiotic-Supplemented Diet		SEM	*p*-Value		
	CP Level (%)		CP Level (%)					
	18	24	18	24		CP	ANT	INT
Water deposition rate, g/d	196.82	262.28	226.01	263.85	7.95	<0.001	0.156	0.200
Protein deposition rate, g/d	42.27 ^c^	65.25 ^a^	53.83 ^b^	59.46 ^ab^	2.22	<0.001	0.234	0.002
Lipid deposition rate, g/d	28.45	26.20	29.24	21.32	0.95	0.002	0.154	0.054
Ash deposition rate, g/d	8.49	9.11	9.45	9.13	0.21	0.724	0.261	0.284
Gross energy deposition rate, MJ/d	2.08 ^c^	2.60 ^a^	2.53 ^ab^	2.22 ^bc^	0.07	0.355	0.773	0.002

Abbreviations: CP—main effect of crude protein; ANT—main effect of antibiotic; INT—interaction effect between crude protein and antibiotic. Statistical significance was declared at *p* < 0.05, and tendencies were declared at 0.05 < *p* < 0.10. Data were subjected to two-way ANOVA. When the interaction was significant, treatment means were compared using Duncan’s multiple range test. ^a,b,c^ Means within a row with different superscripts differ significantly (*p* < 0.05).

**Table 6:** Effects of S1 phase diets with or without antibiotics and CP level on subsequent body composition deposition rates of piglets.

Item	Antibiotic-Free Diet		Antibiotic-Supplemented Diet		SEM	*p*-Value		
	CP Level (%)		CP Level (%)					
	18	24	18	24		CP	ANT	INT
Water deposition rate, g/d	375.58	344.97	365.32	340.64	7.57	0.085	0.633	0.846
Protein deposition rate, g/d	95.38	85.74	88.22	84.3	1.92	0.082	0.254	0.443
Lipid deposition rate, g/d	60.07	53.47	54.94	68.46	1.87	0.196	0.074	0.001
Ash deposition rate, g/d	13.44	12.48	13.05	13.11	0.23	0.374	0.814	0.311
Gross energy deposition rate, MJ/d	4.75 ^b^	4.17 ^b^	4.14 ^b^	4.79 ^a^	0.11	0.841	0.981	0.006

Abbreviations: CP—main effect of crude protein; ANT—main effect of antibiotic; INT—interaction effect between crude protein and antibiotic. Statistical significance was declared at *p* < 0.05, and tendencies were declared at 0.05 < *p* < 0.10. Data were subjected to two-way ANOVA. When the interaction was significant, treatment means were compared using Duncan’s multiple range test. ^a,b^ Means within a row with different superscripts differ significantly (*p* < 0.05).

**Table 7:** Effects of S1 phase diets with or without antibiotics and CP level on whole experimental body composition deposition rates of piglets.

Item	Antibiotic-Free Diet		Antibiotic-Supplemented Diet		SEM	*p*-Value		
	CP Level (%)		CP Level (%)					
	18	24	18	24		CP	ANT	INT
Whole experimental period (0 d~25 kg BW)								
Water deposition rate, g/d	315.46	317.09	318.6	313.86	5.00	0.89	0.997	0.777
Protein deposition rate, g/d	77.33	78.84	76.67	75.66	1.23	0.927	0.48	0.642
Lipid deposition rate, g/d	49.37 ^ab^	44.3 ^b^	46.28 ^b^	52.14 ^a^	1.09	0.823	0.194	0.007
Ash deposition rate, g/d	11.81	11.34	11.86	12.51	0.22	0.84	0.186	0.22
Gross energy deposition rate, MJ/d	3.84	3.64	3.6	3.9	0.07	0.702	0.953	0.084

Abbreviations: CP—main effect of crude protein; ANT—main effect of antibiotic; INT—interaction effect between crude protein and antibiotic. Statistical significance was declared at *p* < 0.05, and tendencies were declared at 0.05 < *p* < 0.10. Data were subjected to two-way ANOVA. When the interaction was significant, treatment means were compared using Duncan’s multiple range test. ^a,b^ Means within a row with different superscripts differ significantly (*p* < 0.05).

**Table 8:** Effects of CP level on serum biochemical parameters of weaned piglets fed diets with or without antibiotics.

Item	Antibiotic-Free Diet		Antibiotic-Supplemented Diet		SEM	*p*-Value		
	CP Level (%)		CP Level (%)					
	18	24	18	24		CP	ANT	INT
Total protein, g/L	52.77	48.99	50.16	48.19	0.83	0.088	0.295	0.575
Albumin, g/L	27.53	27.37	28.83	26.51	0.69	0.404	0.882	0.467
Blood urea nitrogen, mmol/L	5.47	8.52	5.08	8.52	0.56	0.003	0.831	0.835
Glucose, mmol/L	8.04	6.48	7.63	6.97	0.30	0.083	0.95	0.457
Triglyceride, mmol/L	0.66	0.52	0.55	0.50	0.02	0.012	0.090	0.251
Cholesterol, mmol/L	2.25	2.09	2.20	2.00	0.05	0.106	0.528	0.806
Alanine aminotransferase, U/L	50.91	49.70	53.63	46.30	1.57	0.197	0.916	0.349
Aspartate aminotransferase, U/L	57.57	45.30	44.85	44.36	2.40	0.170	0.144	0.204
Alkaline phosphatase, U/L	500.99	438.93	529.48	554.46	38.48	0.822	0.387	0.599
Creatinine, μmol/L	76.59	77.68	76.02	82.76	2.43	0.456	0.666	0.588

Abbreviations: CP—main effect of crude protein; ANT—main effect of antibiotic; INT—interaction effect between crude protein and antibiotic. Statistical significance was declared at *p* < 0.05, and tendencies were declared at 0.05 < *p* < 0.10.

**Table 9:** Effects of S1 phase diets with or without antibiotics and CP level on serum biochemical parameters of piglets at 25 kg BW.

Item	Antibiotic-Free Diet		Antibiotic-Supplemented Diet		SEM	*p*-Value		
	CP Level (%)		CP Level (%)					
	18	24	18	24		CP	ANT	INT
Total protein, g/L	54.65	54.55	52.95	55.98	0.73	0.348	0.927	0.316
Albumin, g/L	30.93	29.70	28.88	30.62	0.48	0.791	0.564	0.142
Blood urea nitrogen, mmol/L	8.66	7.81	10.56	8.67	0.65	0.312	0.310	0.701
Glucose, mmol/L	4.42	4.07	3.29	4.66	0.26	0.325	0.589	0.102
Triglyceride, mmol/L	0.50	0.54	0.51	0.45	0.03	0.892	0.549	0.412
Cholesterol, mmol/L	2.04	1.97	2.18	2.15	0.06	0.703	0.203	0.837
Alanine aminotransferase, U/L	63.92	55.64	65.66	62.35	3.23	0.413	0.548	0.722
Aspartate aminotransferase, U/L	83.51	70.56	63.06	69.42	4.58	0.732	0.271	0.323
Alkaline phosphatase, U/L	266.79	285.41	291.71	218.39	13.83	0.317	0.439	0.102
Creatinine, μmol/L	79.98	80.21	76.93	83.1	1.98	0.461	0.985	0.493

Abbreviations: CP—main effect of crude protein; ANT—main effect of antibiotic; INT—interaction effect between crude protein and antibiotic. Statistical significance was declared at *p* < 0.05, and tendencies were declared at 0.05 < *p* < 0.10.

**Table 10:** Effects of CP level on nutrient apparent digestibility of weaned piglets fed diets with or without antibiotics.

Item	Antibiotic-Free Diet		Antibiotic-Supplemented Diet		SEM	*p*-Value		
	CP Level (%)		CP Level (%)					
	18	24	18	24		CP	ANT	INT
Dry matter	83.04	83.20	82.34	82.24	0.26	0.955	0.134	0.808
CP	74.60	77.50	74.46	75.36	0.54	0.076	0.273	0.334
Ether extract	84.42	80.98	83.14	84.86	0.70	0.525	0.341	0.069
Ash	59.88	61.42	58.06	58.74	0.54	0.275	0.036	0.667
Gross energy	83.96	84.02	83.12	83.12	0.27	0.958	0.136	0.958

Abbreviations: CP—main effect of crude protein; ANT—main effect of antibiotic; INT—interaction effect between crude protein and antibiotic. Statistical significance was declared at *p* < 0.05, and tendencies were declared at 0.05 < *p* < 0.10.

**Table 11:** Effects of S1 phase diets with or without antibiotics and CP level on subsequent nutrient apparent digestibility of piglets.

Item	Antibiotic-Free Diet		Antibiotic-Supplemented Diet		SEM	*p*-Value		
	CP Level (%)		CP Level (%)					
	18	24	18	24		CP	ANT	INT
Dry matter	86.44	87.24	86.03	86.64	0.19	0.059	0.163	0.793
CP	81.34	83.66	79.38	82.60	0.47	0.010	0.031	0.490
Ether extract	77.20	73.44	74.00	74.70	0.70	0.275	0.484	0.119
Ash	53.40	56.30	55.68	54.46	0.66	0.534	0.870	0.138
Gross energy	87.20	88.20	86.16	87.30	0.23	0.006	0.011	0.838

Abbreviations: CP—main effect of crude protein; ANT—main effect of antibiotic; INT—interaction effect between crude protein and antibiotic. Statistical significance was declared at *p* < 0.05, and tendencies were declared at 0.05 < *p* < 0.10.

**Table 12:** Effects of dietary CP level on serum free AA concentration of weaned piglets fed diets with or without antibiotics.

Item	Antibiotic-Free Diet		Antibiotic-Supplemented Diet		SEM	*p*-Value		
	CP Level (%)		CP Level (%)					
	18	24	18	24		CP	ANT	INT
Aspartic acid	0.019	0.014	0.019	0.023	0.002	0.852	0.236	0.166
Threonine	0.368	0.272	0.449	0.270	0.023	0.001	0.268	0.242
Serine	0.164	0.155	0.155	0.181	0.007	0.519	0.541	0.209
Asparagine	0.056	0.060	0.066	0.060	0.003	0.870	0.489	0.505
Glutamic acid	0.121	0.110	0.113	0.138	0.006	0.583	0.459	0.191
Glutamine	0.234	0.263	0.284	0.204	0.018	0.503	0.909	0.166
Glycine	0.671 ^b^	0.692 ^b^	0.909 ^a^	0.676 ^b^	0.028	0.009	0.007	0.003
Alanine	0.748	0.624	0.757	0.739	0.027	0.189	0.243	0.314
Valine	0.364	0.171	0.42	0.173	0.029	<0.001	0.262	0.299
Methionine	0.033	0.032	0.029	0.028	0.002	0.792	0.297	0.842
Isoleucine	0.104	0.048	0.122	0.053	0.013	0.019	0.620	0.786
Leucine	0.150	0.151	0.172	0.169	0.007	0.938	0.195	0.883
Tyrosine	0.092	0.110	0.113	0.121	0.005	0.240	0.155	0.671
Phenylalanine	0.062	0.063	0.073	0.070	0.002	0.860	0.05	0.681
Lysine	0.365	0.196	0.434	0.214	0.029	<0.001	0.254	0.492
Histidine	0.015	0.040	0.020	0.030	0.004	0.062	0.741	0.381
Arginine	0.068	0.096	0.096	0.070	0.006	0.930	0.917	0.044

Abbreviations: CP—main effect of crude protein; ANT—main effect of antibiotic; INT—interaction effect between crude protein and antibiotic. Statistical significance was declared at *p* < 0.05, and tendencies were declared at 0.05 < *p* < 0.10. Data were subjected to two-way ANOVA. When the interaction was significant, treatment means were compared using Duncan’s multiple range test. ^a,b^ Means within a row with different superscripts differ significantly (*p* < 0.05).

**Table 13:** Effects of early diets with or without antibiotics and CP level on subsequent serum AA concentration of piglets at 25 kg BW.

Item	Antibiotic-Free Diet		Antibiotic-Supplemented Diet		SEM	*p*-Value		
	CP Level (%)		CP Level (%)					
	18	24	18	24		CP	ANT	INT
Aspartic acid	0.020	0.022	0.018	0.014	0.002	0.776	0.212	0.452
Threonine	0.174	0.197	0.157	0.156	0.009	0.562	0.132	0.500
Serine	0.156	0.124	0.148	0.119	0.005	0.001	0.370	0.839
Asparagine	0.058	0.043	0.051	0.048	0.003	0.118	0.872	0.316
Glutamic acid	0.190	0.204	0.171	0.178	0.016	0.755	0.505	0.911
Glutamine	0.462	0.378	0.441	0.410	0.025	0.280	0.915	0.610
Glycine	1.116	1.057	1.044	1.082	0.054	0.932	0.840	0.680
Alanine	0.554	0.545	0.457	0.449	0.029	0.892	0.121	0.988
Valine	0.219	0.233	0.233	0.182	0.016	0.580	0.572	0.337
Methionine	0.038	0.053	0.043	0.032	0.004	0.791	0.249	0.066
Isoleucine	0.098	0.107	0.110	0.078	0.007	0.378	0.518	0.130
Leucine	0.166	0.181	0.183	0.131	0.011	0.411	0.442	0.130
Tyrosine	0.084	0.079	0.081	0.069	0.006	0.506	0.614	0.782
Phenylalanine	0.094	0.095	0.086	0.072	0.006	0.620	0.223	0.568
Lysine	0.195	0.248	0.209	0.178	0.014	0.696	0.316	0.135
Histidine	0.065	0.062	0.046	0.062	0.005	0.497	0.337	0.318
Arginine	0.117	0.133	0.128	0.101	0.006	0.670	0.424	0.101

Abbreviations: CP—main effect of crude protein; ANT—main effect of antibiotic; INT—interaction effect between crude protein and antibiotic. Statistical significance was declared at *p* < 0.05, and tendencies were declared at 0.05 < *p* < 0.10.

**Table 14:** Effects of CP level on intestinal morphology of weaned piglets fed diets with or without antibiotics.

Item	Antibiotic-Free Diet		Antibiotic-Supplemented Diet		SEM	*p*-Value		
	CP Level (%)		CP Level (%)					
	18	24	18	24		CP	ANT	INT
Duodenum, μm								
Villus height	584.51	623.32	644.28	579.66	18.49	0.738	0.835	0.192
Crypt depth	249.75	215.60	208.32	219.16	9.52	0.552	0.338	0.258
Villus height/crypt ratio	2.38	3.06	3.15	2.73	0.16	0.678	0.48	0.089
Jejunum, μm								
Villus height	486.17	494.35	443.41	427.96	19.41	0.929	0.19	0.771
Crypt depth	183.10	178.37	188.32	177.60	5.19	0.497	0.844	0.791
Villus height/crypt ratio	2.71	2.77	2.43	2.39	0.11	0.949	0.171	0.828
Ileum, μm								
Villus height	340.22	365.95	340.53	314.12	11.21	0.988	0.272	0.267
Crypt depth	187.62	160.68	174.08	187.43	8.26	0.697	0.705	0.257
Villus height/crypt ratio	1.83	2.32	2.11	1.75	0.12	0.776	0.536	0.079

Abbreviations: CP—main effect of crude protein; ANT—main effect of antibiotic; INT—interaction effect between crude protein and antibiotic. Statistical significance was declared at *p* < 0.05, and tendencies were declared at 0.05 < *p* < 0.10.

**Table 15:** Effects of CP level on pH of gastrointestinal contents of weaned piglets fed diets with or without antibiotics.

Item	Antibiotic-Free Diet		Antibiotic-Supplemented Diet		SEM	*p*-Value		
	CP Level (%)		CP Level (%)					
	18	24	18	24		CP	ANT	INT
Stomach	3.4	4.23	3.18	3.53	0.18	0.099	0.193	0.482
Duodenum	5.73	5.91	5.79	5.68	0.08	0.835	0.621	0.41
Jejunum	5.72	5.54	5.86	5.9	0.10	0.743	0.247	0.607
Ileum	6.33	6.53	6.21	6.62	0.11	0.182	0.939	0.639
Colon	6.31	6.24	6.5	6.16	0.07	0.148	0.684	0.348

Abbreviations: CP—main effect of crude protein; ANT—main effect of antibiotic; INT—interaction effect between crude protein and antibiotic. Statistical significance was declared at *p* < 0.05, and tendencies were declared at 0.05 < *p* < 0.10.

## Data Availability

The data presented in this study are available upon request from the corresponding author.

## Abbreviations

The following abbreviations are used in this manuscript:

ADG	Average daily gain
ADFI	Average daily feed intake
ALB	Albumin
ALT	Alanine aminotransferase
AST	Aspartate aminotransferase
ALP	Alkaline phosphatase
BUN	Blood urea nitrogen
CHO	Cholesterol
CRE	Creatinine
CP	Crude protein
GLU	Glucose
G:F	Gain to feed
TG	Triglyceride
TP	Total protein
